# Enhanced bone assessment of the shoulder using zero-echo time MRI with deep-learning image reconstruction

**DOI:** 10.1007/s00256-024-04690-8

**Published:** 2024-04-24

**Authors:** Falko Ensle, Malwina Kaniewska, Maelene Lohezic, Roman Guggenberger

**Affiliations:** 1 Diagnostic and Interventional Radiology, University Hospital Zurich, University Zurich, Zurich, Switzerland; 2 University of Zurich (UZH), Raemistrasse 100, CH-8091 Zurich, Switzerland; 3 GE HealthCare, Zurich, Switzerland

**Keywords:** CT-like MRI, Zero echo time, Deep learning, Shoulder, Bone

## Abstract

**Objectives:**

To assess a deep learning-based reconstruction algorithm (DLRecon) in zero echo-time (ZTE) MRI of the shoulder at 1.5 Tesla for improved delineation of osseous findings.

**Methods:**

In this retrospective study, 63 consecutive exams of 52 patients (28 female) undergoing shoulder MRI at 1.5 Tesla in clinical routine were included. Coronal 3D isotropic radial ZTE pulse sequences were acquired in the standard MR shoulder protocol. In addition to standard-of-care (SOC) image reconstruction, the same raw data was reconstructed with a vendor-supplied prototype DLRecon algorithm. Exams were classified into three subgroups: no pathological findings, degenerative changes, and posttraumatic changes, respectively. Two blinded readers performed bone assessment on a 4-point scale (0-poor, 3-perfect) by qualitatively grading image quality features and delineation of osseous pathologies including diagnostic confidence in the respective subgroups. Quantitatively, signal-to-noise ratio (SNR) and contrast-to-noise ratio (CNR) of bone were measured. Qualitative variables were compared using the Wilcoxon signed‐rank test for ordinal data and the McNemar test for dichotomous variables; quantitative measures were compared with Student’s *t*-testing.

**Results:**

DLRecon scored significantly higher than SOC in all visual metrics of image quality (all, *p* < 0.03), except in the artifact category (*p* = 0.37). DLRecon also received superior qualitative scores for delineation of osseous pathologies and diagnostic confidence (*p* ≤ 0.03). Quantitatively, DLRecon achieved superior CNR (95 CI [1.4–3.1]) and SNR (95 CI [15.3–21.5]) of bone than SOC (*p* < 0.001).

**Conclusion:**

DLRecon enhanced image quality in ZTE MRI and improved delineation of osseous pathologies, allowing for increased diagnostic confidence in bone assessment.

## Introduction

Shoulder MRI is the preferred imaging modality for diagnostic workup of a wide spectrum of clinically suspected pathologies, e.g., degenerative disease or traumatic injuries [1]. In particular, MRI provides an excellent assessment of soft tissues and bone marrow. Additionally, even detailed evaluation of mineralized bone or other calcified structures with ultrashort T2 relaxation properties has become feasible through zero echo time (ZTE) MR imaging with “CT-like” bone contrast [2–4]. Recent introduction of commercially available software has facilitated routine implementation of this technique into standard-of-care MR protocols [5–8]. Benefits of ZTE application in shoulder MRI were previously demonstrated, with strong inter-modality agreements between CT and ZTE images [9–11]. As an inherently 3D volume technique, ZTE also accommodates multiplanar reformations and maximum intensity projection renderings, like CT.

However, overall bone depiction quality with ZTE MRI is still inferior to that of CT, despite continued development. Optimization of ZTE sequences remains challenging as it entails a trade-off between spatial resolution, signal-to-noise ratio (SNR), and scan time [5]. Short-T2 imaging is generally more SNR-limited than conventional MRI due to its sub-millisecond and high-bandwidth acquisitions [12]. This also implies certain hardware requirements in terms of radiofrequency chains and gradient performance [13]. Recent research to improve SNR efficiency within clinically reasonable scan times has focused on methods for retrieving central k-space data missed during the dead-time gaps arising from radio-frequency excitation and switching in ZTE imaging [14]. However, inherent limitations of conventional image reconstruction persist.

The advent of artificial intelligence (AI) in image reconstruction offers a novel approach to address these challenges in ZTE MRI [15]. Deep learning reconstruction (DLRecon) algorithms have been shown to be capable of decoupling the traditional SNR interdependence, i.e., to improve image quality and reduce scan time simultaneously [16]. Commercial DLRecon implementations are being increasingly incorporated into clinical protocols across a growing range of MRI techniques, while promising results have also been reported in the shoulder [17, 18]. Applying DLRecon to ZTE shoulder MRI could further enhance its diagnostic yield in osseous findings within reasonable scan times. Consequently, the AI-enhanced ZTE sequence could be more widely established as a routine component of shoulder MRI, which then may even serve as a one-stop modality for some patients, obviating additional CT together with its radiation exposure and further costs.

We hypothesized that DLRecon would enhance the overall image quality of ZTE sequence in the shoulder, enabling increased diagnostic confidence in the clinical evaluation of osseous structures including pathologies. The objective of our study was to assess the efficacy of a DLRecon algorithm to improve both image quality and bone evaluation in ZTE shoulder MRI, compared to standard-of-care (SOC) reconstruction.

## Material and methods

### Study design

This study was approved by the institutional review board. Written general consent was obtained from all participants prior to imaging.

In this retrospective study, 67 consecutive shoulder exams of 56 patients were included between July and November 2022. Three exams were not reconstructed with the DL algorithm by the scanner. Twelve patients had scans of both shoulders, one of them was analyzed in the same subgroup and therefore only the right-sided exam of that individual was included to avoid dependent data. This resulted in *n* = 63 exams of 52 patients for final analysis. Fourteen exams were performed as MR arthrographies.

Clinical indications for MRI included a history of shoulder trauma (*n* = 21), chronic rotator cuff pathology (*n* = 14), glenohumeral instability (*n* = 10), unspecific shoulder pain (*n* = 9), postoperative setting (*n* = 4), frozen shoulder (*n* = 3), bursitis (*n* = 2), and rheumatoid arthritis (*n* = 1).

All exams were classified into three subgroups according to the written radiology report: (1) no pathology, (2) degenerative changes, and (3) posttraumatic changes. Demographics of each subgroup are shown in Table 1.

**Table 1:** Study subgroups

Characteristic	No pathology	Degenerative changes	Posttraumatic changes
Examinations (*n*)	23	25	15
Age (years)	43 ± 14	57 ± 9	42 ± 16
Males/females	12/11	12/13	11/4
Osseous pathology (*n*)	−	Acromioclavicular osteoarthritis (14); glenohumeral osteoarthritis (11)	Hill-Sachs lesion (5); greater tuberosity fracture (3); osseous Bankart lesion (2); AC-joint injury (2); clavicle fracture (1); humeral head subchondral fracture (1); scapula fracture (1)

All patients who presented to our institution for clinically indicated MR shoulder were considered for study inclusion. Exclusion criteria were the inability to retrospectively reconstruct with DLRecon due to incomplete local storage of the raw data at the time of acquisition and patient age < 18 years. Furthermore, exams with severely degraded overall image quality on the standard reconstruction due to hardware or motion artifacts would be excluded from the analysis.

### Image acquisition

All examinations were performed on the same clinical 1.5 Tesla MRI System (SIGNA™ Artist, GE HealthCare, Waukesha, WI, USA). Patients were scanned in a supine position using a dedicated 16-channel shoulder coil. Coronal 3D isotropic radial ZTE pulse sequence was obtained as part of the institution’s standard MR shoulder protocol [19]. Scan parameters are listed in Table 2.

**Table 2:** Parameters of ZTE MR sequence

Parameter	Value
Echo time (ms)	≅ 0
Repetition time (ms)	401.71
Flip angle (°)	1
Pixel bandwidth (kHz)	488
Number of excitations	4
Field of view (cm)	18
Slice thickness (mm, 0.6 mm gap)	1.2
Acquisition matrix	172 × 172
Scan time (min)	~ 2:23

### Deep learning image reconstruction

Standard reconstruction for the ZTE sequence by the scanner’s native, inline reconstruction pipeline was performed in routine clinical practice and is termed standard-of-care (SOC) in this study [20]. Additionally, the acquired raw data was reconstructed with a vendor-supplied prototype of a deep learning-based reconstruction pipeline (AIR™ Recon DL, GE HealthCare, Waukesha, WI, USA). DLRecon includes a deep convolutional neural network (CNN) that operates on raw k-space data to provide sharp, low-noise images [21]. Specifically, DLRecon is designed to reduce image noise and truncation artifacts while improving edge sharpness to enhance image quality. The CNN was trained in a supervised learning approach using diverse pairs of near-perfect MR images and synthesized lower-quality versions with more truncation artifacts and higher noise levels. The training database included four million unique image combinations spanning a broad range of image content to enable generalizability across all anatomies. The prototype allows a user-tunable noise reduction factor ranging from 0 to 100%. In advance, a sample set of exams not included in the study was reconstructed with varying denoising levels and reviewed by the authors. For the present study, the highest denoising level (100%) was activated in order to investigate the biggest difference between the reconstruction methods and to detect potential blurring or thresholding of image details. Denoising is controlled independently of the ringing reduction and does not affect edge sharpness to preserve image features [22]. The tested prototype is an extension of the commercially available version of DLRecon, making it compatible with 3D ZTE data.

### Image analysis

#### Quantitative analysis

To quantitatively assess image quality, signal-to-noise ratio (SNR) and contrast-to-noise ratio (CNR) of bone were measured in both reconstruction methods. The subset with fractures was excluded from quantitative analysis to avoid potential distortion. Furthermore, to avoid dependent data, the left shoulder exam was excluded in five patients with scans of both shoulders, resulting in *n* = 43 exams for the final analysis.

Five-millimeter squared regions of interest (ROI) were placed on the same single axial slice at mid-glenoid level in the following locations: (1) central humeral head and glenoid as a reference for fine- and coarse-structured spongy bone, respectively, and (2) subscapular muscle next to the glenoid.

Additionally, the cortical bone of the proximal humeral shaft was measured in the first proximal slice that could accommodate a 3 mm^2^ ROI. Mean and standard deviation (SD) values of signal intensity (SI) were calculated for each ROI. The mean of both bone and muscle SD values was computed to obtain estimates of noise from different locations inside the anatomy.

The mean of both spongy and cortical bone measurements served as bone signal for the SNR and CNR, which were calculated as follows:

$$SNR= \frac{SI\;bone}{mean\;SD\;of\;bone\;and\;muscle}$$

$$CNR= \frac{SI\;bone-SI\;muscle}{mean\;SD\;of\;bone\;and\;muscle}$$

#### Qualitative analysis

ZTE sequences were analyzed independently by two readers (a board-certified radiologist with 8 years and a radiology resident with 4 years of experience) on standard PACS workstations (DeepUnity Diagn, Dedalus, Bonn, Germany) with diagnostic quality monitors. Both readers were blinded to the reconstruction method, clinical information, and radiological report. All scans were randomized for evaluation of the entire imaging volume in all three planes using a multiplanar reconstruction tool in PACS. Images were gray-scale inverted for CT-like visualization. To establish consistency, observers underwent a prior training session by jointly reviewing a separate practice set of 10 exams until consensus was reached.

The qualitative scoring system is summarized in Table 3. A 4-point scale (0-poor/non-diagnostic, 1-acceptable, 2-good, 3-perfect) was used to visually score image quality attributes. In the subset without pathology, image quality was assessed by grading delineation of both cortical and trabecular bone, anatomic conspicuity, and overall image quality. In the group with degenerative changes, delineation of osteophytosis, subchondral cysts, subchondral sclerosis, and soft tissue calcifications were separately evaluated, if present. The subset with fractures was graded for delineation of fracture lines and osseous fragments, while displacement was scored dichotomously (yes/no). Additionally, reviewers rated their diagnostic confidence in visualizing degeneration and fractures in the respective subsets with pathology.

**Table 3:** Summary of 4-point qualitative scoring system for assessing image quality and pathologies

Score	0	1	2	3
Delineation of cortical and trabecular bone	Substantial obscuration of structures	Impaired delineation of structural detail	Mostly continuous and sharp visualization	Continuous and sharp visualization
Anatomic conspicuity	Barely apparent	Mostly discernible	Well visible	Very conspicuous
Overall image quality	Poor SNR/CNR, non-definable contours	Fair SNR/CNR, slightly blurred contours	Good SNR/CNR, mostly sharp contours	Perfect SNR/CNR, ubiquitously sharp contours
Artifacts	None	No detrimental effect on diagnostic yield	Assessment partially limited, still diagnostic	Diagnostic evaluation not feasible
Delineation of pathology	Non-diagnostic visualization	Partial visualization	Almost full visualization	Detailed visualization
Diagnostic confidence	None	Low confidence	Intermediate confidence	High confidence

The presence of artifacts in the group without pathology was graded on a 4-point scale (0-none, 1-mild, 2-moderate, 3-severe), regarding their detrimental effect on the depiction of osseous structures.

### Statistical analysis

The Shapiro–Wilk test was used to assess the normality of data. Normally distributed variables are reported as mean ± SD and non-normally distributed variables are reported as median ± interquartile range (IQR). Reading scores of qualitative image analysis were compared using the non-parametric paired sample Wilcoxon signed‐rank test for ordinal data and the McNemar test for dichotomous variables. Quantitative analysis was evaluated by paired sample Student’s *t*-testing and reported with mean difference and 95% confidence intervals (95 CI).

Inter-rater agreement was calculated through linearly weighted Cohen’s kappa with 95% confidence intervals (95 CI) and with values being interpreted as follows: 0.00–0.20 = poor agreement, 0.21–0.40 = fair agreement, 0.41–0.60 = moderate agreement, 0.61–0.80 = substantial agreement, 0.81–1.00 = (almost) perfect agreement.

*p*-values below 0.05 were considered significant. All calculations were performed in SPSS (IBM SPSS Statistics, version 29.0; IBM, Armonk, NY, USA).

## Results

The final study cohort consisted of 63 exams in 52 patients (mean age: 46 ± 14 years, 28 females). Detailed patient demographics are listed in Table 1.

### Quantitative analysis

**Fig. 1:**
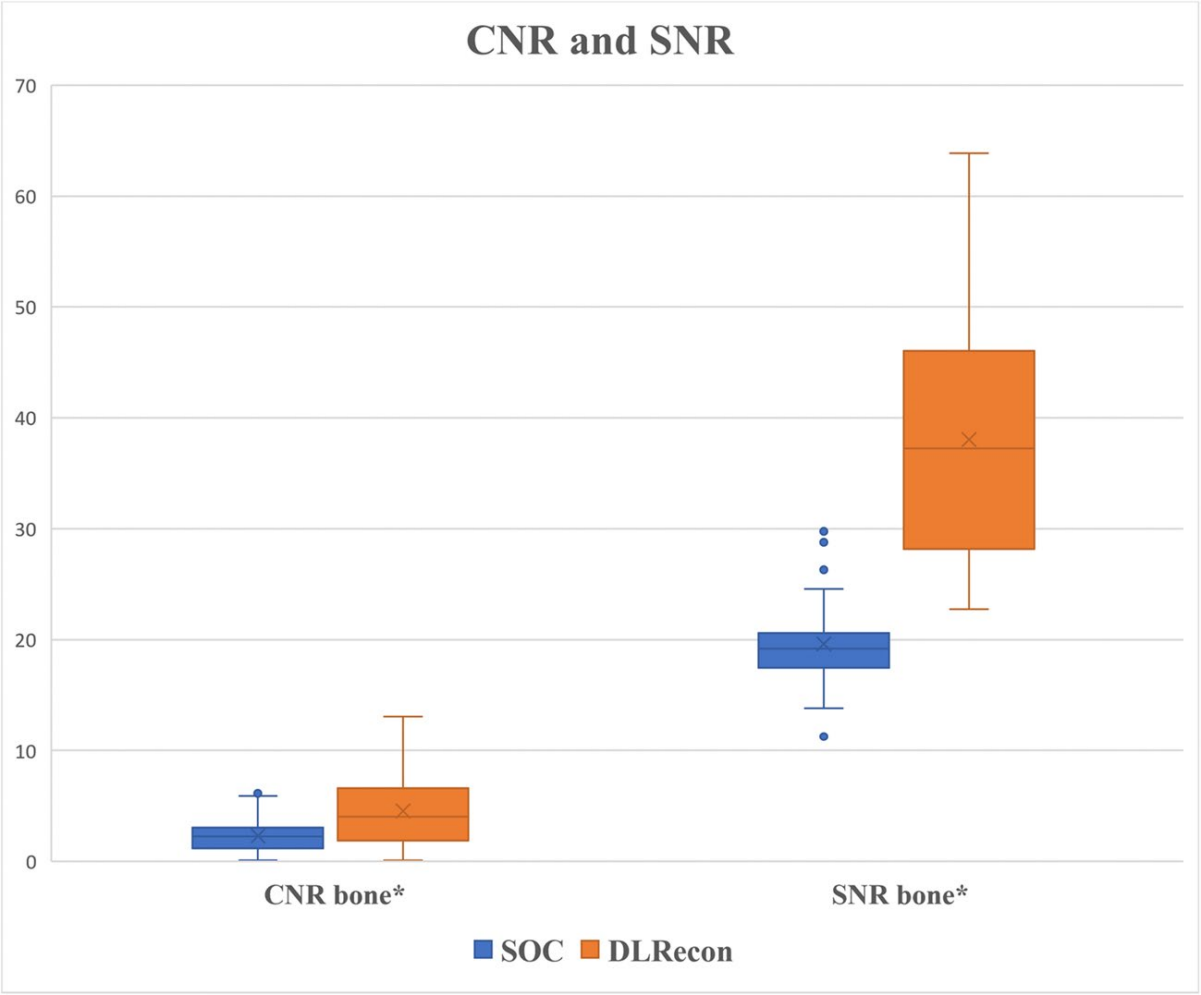
Box and whisker plot comparing CNR and SNR of bone between standard-of-care (SOC) and deep learning reconstruction (DLRecon) method (*mark: *p* < 0.001). Abbreviations: CNR, contrast-to-noise ratio; SNR, signal-to-noise ratio

DLRecon achieved significantly higher CNR and SNR of bone compared to SOC, as illustrated in Fig. 1. Mean ± SD of CNR from SOC and DLRecon were 2.3 ± 1.4 and 4.5 ± 3.2 (mean difference: 2.2); SNR were 19.6 ± 3.7 and 38.0 ± 9.7 (mean difference: 18.4). The *p*-value for CNR (95 CI [1.4–3.1]) and SNR (95 CI [15.3–21.5]) comparisons was less than 0.001. DLRecon showed greater variability of CNR and SNR measurements compared to SOC, which has also been observed in previous studies of the same underlying DLRecon method [23, 24].

### Qualitative analysis

**Fig. 2:**
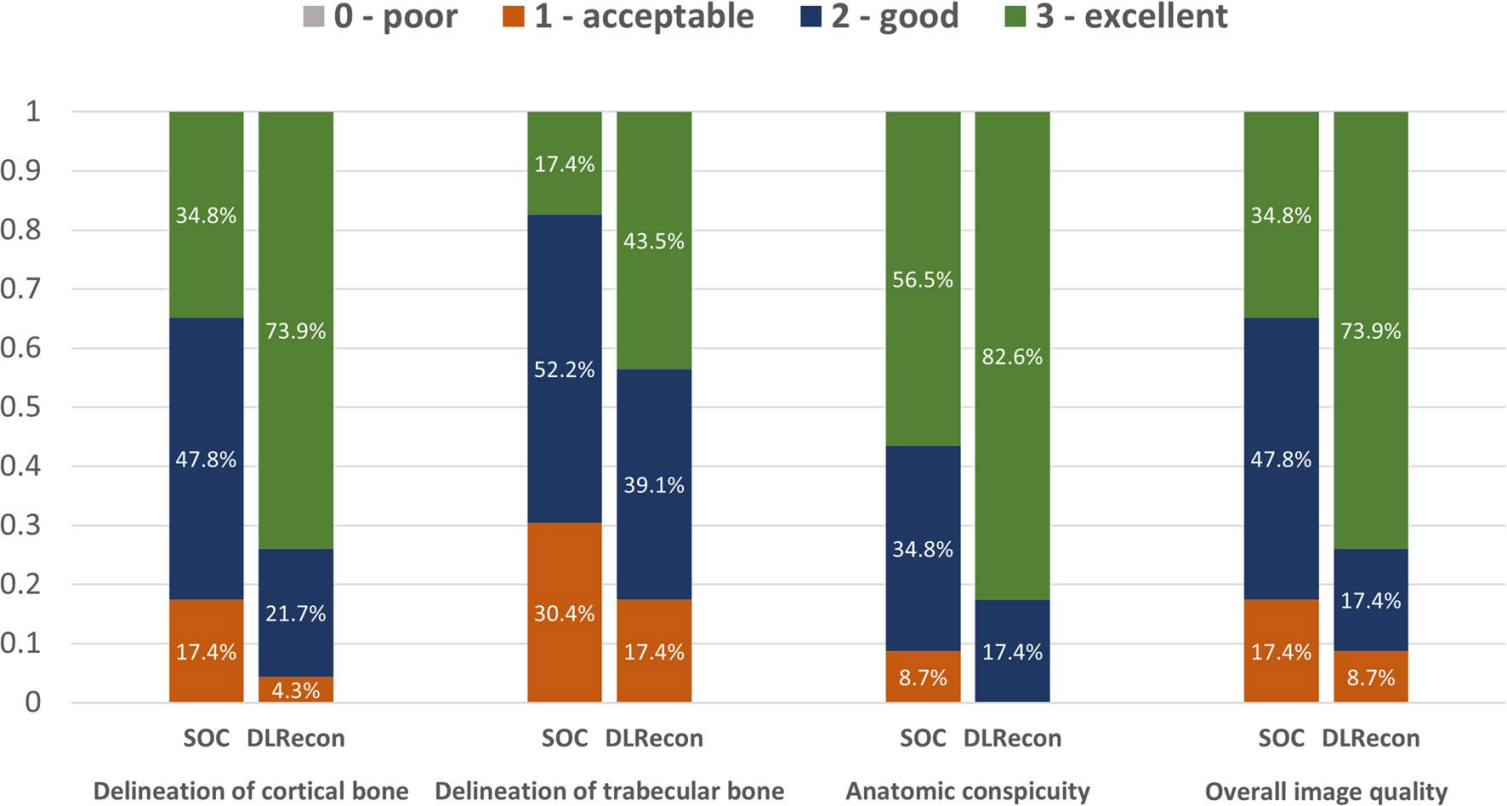
Graphic overview of visual scores regarding image quality in the subgroup with no pathology (*n* = 23). Abbreviations: DLRecon, deep learning reconstruction; SOC, standard-of-care

**Fig. 3:**
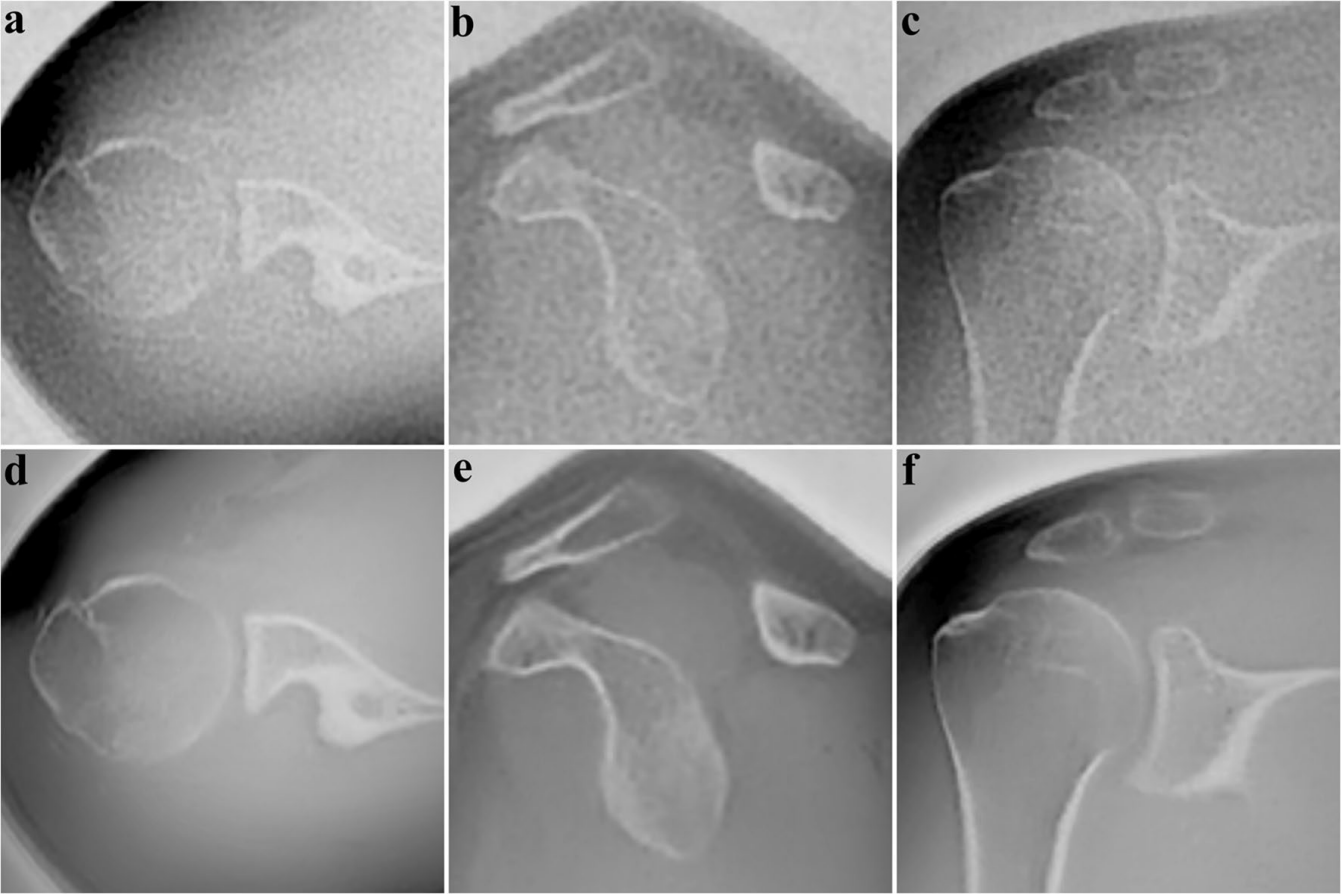
Twenty-nine-year-old male presenting after shoulder trauma. Axial, sagittal, and coronal reformats of the ZTE sequence processed with DLRecon (**d, e, f**) exhibit superior image quality due to denoising and sharpening, compared to the same images from SOC reconstruction (**a, b, c**). No osseous abnormality was identified on this exam

The distribution of image quality features evaluated on SOC and DLRecon by the senior reader is demonstrated in Fig. 2. Comparison of median scores (IQR) with associated* p*-values and inter-rater agreement for all subgroups is shown in Table 4. DLRecon scored significantly higher than SOC in all image quality features (*p* < 0.03), except in the artifact category, where there was no significant difference between the reconstruction methods (*p* = 0.37). Artifacts were overall scored slightly higher in DLRecon (1 (0–1)) than in SOC (0 (0–1)). Inter-rater agreement for image quality categories ranged from moderate to substantial in both DLRecon (*κ* = 0.52–0.75) and SOC (*κ* = 0.54–0.74). Exemplary images of ZTE bone depiction with DLRecon and SOC are displayed in Fig. 3.

**Table 4:** Comparison of median scores (interquartile range) with associated* p*-values and inter-rater agreement (95% confidence interval) for all subgroups

Metric	Median (IQR)		*p*-value	Inter-rater agreement *κ* [95 CI]	
	SOC	DLRecon		SOC	DLRecon
Image quality					
Delineation of cortical bone	2 (2–3)	3 (2.5–3)	< 0.001	0.61 [0.33–0.89]	0.66 [0.35–0.97]
Delineation of trabecular bone	2 (1–2)	2 (2–3)	0.029	0.54 [0.25–0.83]	0.52 [0.24–0.80]
Anatomic conspicuity	3 (2–3)	3 (3–3)	0.005	0.74 [0.51–0.97]	0.62 [0.21–1.00]
Overall image quality	2 (2–3)	3 (2.5–3)	< 0.001	0.63 [0.35–0.91]	0.75 [0.44–1.00]
Artifacts	0 (0–1)	1 (0–1)	0.366	0.60 [0.32–0.88]	0.60 [0.29–0.91]
Degenerative changes					
Delineation of osteophytosis	2 (1–2)	3 (2–3)	< 0.001	0.76 [0.55–0.97]	0.73 [0.45–1.00]
Delineation of subchondral cysts	2 (1–3)	3 (3–3)	0.001	0.63 [0.35–0.91]	0.63 [0.30–0.96]
Delineation of subchondral sclerosis	2 (2–2)	3 (3–3)	0.002	0.46 [0.04–0.88]	0.57 [0.12–1.00]
Diagnostic confidence	2 (1–2)	3 (2–3)	< 0.001	0.69 [0.45–0.93]	0.61 [0.29–0.93]
Posttraumatic changes					
Delineation of fracture line	2 (2–3)	3 (2.5–3)	0.033	0.76 [0.49–1.00]	0.69 [0.31–1.00]
Delineation of fragments	2 (2–2)	3 (2–3)	0.015	0.63 [0.29–0.97]	0.73 [0.40–1.00]
Diagnostic confidence	2 (2–3)	3 (2.5–3)	0.023	0.56 [0.22–0.90]	0.71 [0.35–1.00]

#### Degenerative changes

Table 5 provides a full summary of the scoring results in the subgroups with degenerative disease and fractures. Delineation of osteophytes, subchondral cysts, and subchondral sclerosis was scored significantly higher with DLRecon compared to SOC (*p* < 0.002). Statistical analysis of soft tissue calcifications was not possible, as it was only present in one exam. This single soft tissue calcification received a good score with SOC and an excellent score in DLRecon. Recently, 3D ZTE was shown to increase the identification of calcific deposits in rotator cuff compared to conventional MR sequences [25].

**Table 5:** Distribution of scoring results assessing the delineation of pathologies and diagnostic confidence in standard-of-care (SOC) and deep learning reconstruction (DLRecon)

	Degenerative changes (*n* = 25)				Posttraumatic changes (*n* = 15)		
Grade	Osteophytes (*n* = 25)	Subchondral cysts (*n* = 21)	Subchondral sclerosis (*n* = 13)	Diagnostic confidence	Fracture line	Fragments	Diagnostic confidence
SOC							
0	1	1	0	1	0	0	0
1	6	5	1	6	3	3	3
2	12	9	12	12	7	9	6
3	6	6	0	6	5	3	6
DLRecon							
0	1	1	0	1	0	0	0
1	1	2	0	1	1	0	0
2	6	2	3	5	3	5	4
3	17	17	10	18	11	10	11

In nearly all exams, degenerative findings of DLRecon and SOC were concordant. In one exam, subchondral cysts were rated to be present with DLRecon, but not in SOC. Subchondral sclerosis was scored as present in one patient each with DLRecon and SOC, but not in the other corresponding reconstruction method.

With regards to overall diagnostic confidence in evaluating degeneration, DLRecon also received significantly higher scores than SOC (*p* < 0.001).

Inter-rater agreement for the delineation of degenerative changes was moderate to substantial for DLRecon (*κ* = 0.57–0.73) and SOC (*κ* = 0.46–0.76). Evaluation of diagnostic confidence demonstrated substantial inter-rater agreement for DLRecon (*κ* = 0.63) and SOC (*κ* = 0.68).

#### Posttraumatic changes

**Fig. 4:**
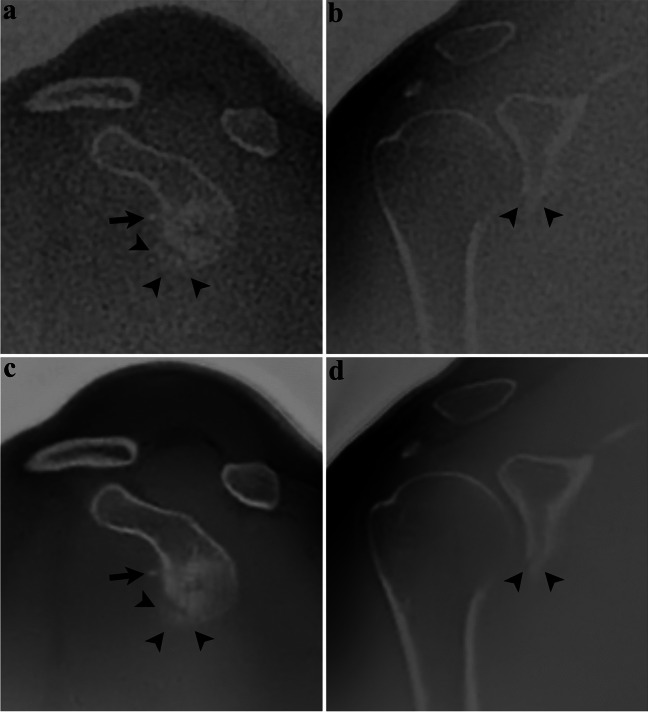
Thirty-nine-year-old man with a history of recurrent shoulder dislocations. Sagittal and coronal ZTE images processed using DLRecon (**c, d**) demonstrate improved delineation of the osseous Bankart lesion (arrowheads), compared to the SOC reconstruction method (**a, b**). Note the small additional osseous fragment anterosuperior to the glenoid (arrow)

Delineation of fracture lines and fragments was significantly improved for DLRecon versus SOC (*p* ≤ 0.03) (Fig. 4). Diagnostic confidence in evaluating these fractures was also rated significantly higher in DLRecon images (*p* = 0.023).

**Fig. 5:**
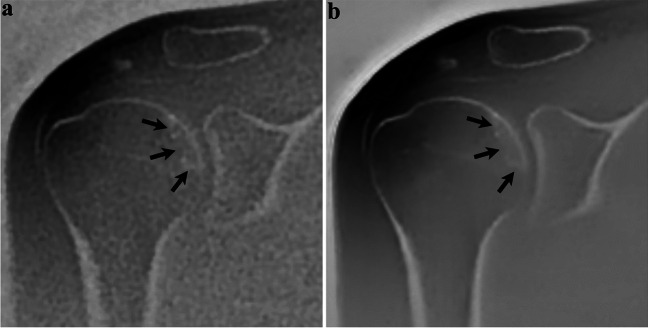
Forty-three-year-old woman with facioscapulohumeral muscular dystrophy, no history of trauma. Coronal ZTE image reconstructed with the SOC method (**a**) shows sclerotic changes (arrows) in the subchondral bone of the humeral head. On the same image processed with DLRecon (**b**), the linear characteristic of sclerosis (arrows) becomes more conspicuous, consistent with a subchondral insufficiency fracture

With regard to dichotomous fracture displacement ratings, there was no statistically significant difference between reconstruction methods (*p* = 1.00) (Fig. 5). Fractures were scored as displaced in 73% of SOC images vs. 80% of DLRecon images. In two patients, the fracture was seen as displaced with DLRecon but not with SOC. In contrast, one fracture was scored as displaced in SOC and non-displaced with DLRecon.

**Fig. 6:**
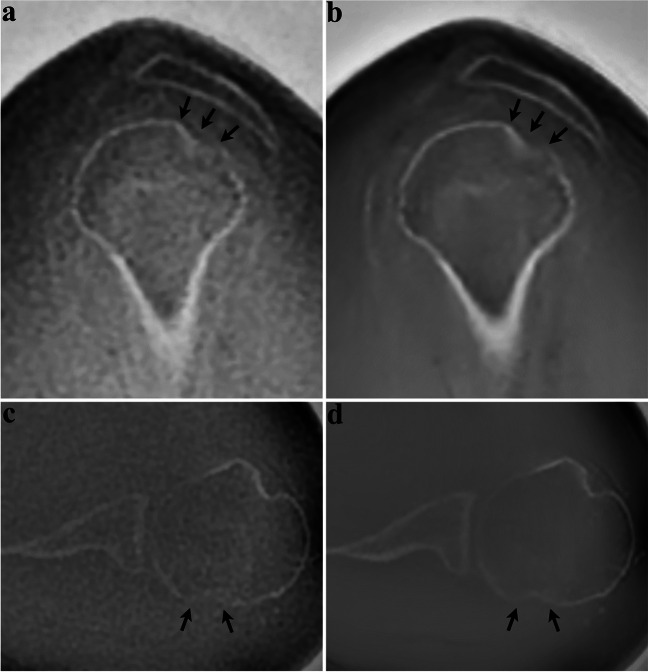
Sixty-seven-year-old woman presenting after shoulder dislocation. Sagittal (upper row) and axial (lower row) ZTE images demonstrate a Hill-Sachs lesion. DLRecon (**b, d**) improves visualization of the depressed cortical fragment (arrows), compared to SOC reconstruction (**a, c**). As a result, the exact extent of the lesion can be assessed with higher diagnostic certainty

Inter-rater agreement for delineation of fracture lines and fragments was substantial for both DLRecon (*κ* = 0.69 and 0.73) and SOC (*κ* = 0.76 and 0.63) (Fig. 6). Inter-rater agreement for diagnostic confidence in evaluating posttraumatic changes was substantial for DLRecon (*κ* = 0.71) and moderate for SOC (*κ* = 0.56).

## Discussion

In this retrospective study, we qualitatively and quantitatively evaluated the efficacy of an AI-reconstruction algorithm (DLRecon) to enhance bone assessment in ZTE MR imaging of the shoulder at 1.5 Tesla. Alongside improvements in overall image quality, DLRecon may improve the delineation of bone including degenerative and posttraumatic changes, compared to SOC reconstruction. Confidently assessing the presence and extent of such osseous findings can be of particular importance in shoulder MRI, e.g., in the setting of shoulder instability and osteoarthritis. DLRecon may also significantly improve diagnostic certainty in the evaluation of osseous abnormalities. These subjective reader results were in line with the objective quantitative metrics of image quality, which suggested superior CNR and SNR of bone.

Our findings, based on a rather small cohort of non-pathological and pathological shoulders, may corroborate the notion that deep-learning reconstruction could add potential clinical value to the ZTE sequence in the shoulder. ZTE imaging provides an accurate depiction of osseous structures with CT-like contrast that is not available with traditional MR pulse sequences. Incorporating a ZTE sequence to the standard shoulder MRI protocol would allow to assess both soft tissue and bony structures precisely by a single MRI scan as an all-in-one diagnostic solution. Such a holistic MRI protocol could further streamline the clinical workflow and may even obviate additional CT with its radiation burden in certain cases. This would be specifically useful in patients with shoulder instability, who often require comprehensive imaging to evaluate soft-tissue stabilizers and osseous support for diagnosis or preoperative planning. In our subset with posttraumatic changes of 15 cases, DLRecon enabled the readers to determine the extent of these osseous lesions more precisely and with greater certainty. These clinically relevant benefits can be attributed to the denoising and sharpening properties of the algorithm, which results in improved conspicuity of cortical bone morphology.

Moreover, DLRecon also improved the delineation of trabecular bone, albeit not to the same level as cortical bone. Visualizing fine trabecular bone structure remains a known shortcoming of ZTE imaging due to the inferior spatial resolution, compared to CT. However, discontinuities of the trabecular structure are clearly depicted, e.g., fracture lines. Further improvement of bone depiction in the deep learning reconstructed ZTE sequence should be achievable in the future with postprocessing by using a signal bias-correction algorithm to optimize bone contrast.

One concern of AI-based reconstruction is compromised image fidelity, i.e., loss of image details or hallucination of new features. However, the detection of clinical findings in the subgroups with degenerative and posttraumatic osseous changes showed an almost perfect concordance between both reconstruction methods. This strong inter-reconstruction agreement descriptively indicates that clinical information was preserved.

To our best knowledge, this is the first study that investigated the clinical application of deep learning reconstruction in ZTE imaging of the shoulder. Our results are concordant with previous research about the DLRecon algorithm in other MR techniques, which also reported superior image quality and enhanced assessment of soft tissue pathology in 2D knee and shoulder MRI [17, 24] as well as 3D MR neurography [23]. For future research, it would be desirable to evaluate the efficacy of DLRecon in ZTE imaging for further anatomic regions and additional osseous pathologies, e.g., erosions in inflammatory rheumatic diseases.

Study limitations include a moderate sample size, particularly in consideration of the individual subgroups. Second, there was no correlation of osseous abnormalities with CT as a reference standard. Our study cohort included a consecutive series of patients in clinical practice, who did not undergo concomitant CT imaging. However, high intermodality agreement between ZTE imaging and CT was reported previously for bone assessment in the shoulder [9, 10]. Third, we applied DLRecon to ZTE MRI at 1.5 Tesla, while it is generally accepted that most MR sequences in musculoskeletal imaging achieve better results at 3 Tesla. Nevertheless, 1.5 Tesla is still widely used in musculoskeletal MRI and could particularly benefit from DLRecon in the quest for shorter scan times and improved image quality. Finally, given the distinct image features of DLRecon, the blinding of readers was unlikely to be fully effective. Despite best precautions, this might have introduced a bias on the scoring, as changes in image smoothness with DLRecon were likely apparent. This effect is accentuated by choosing the highest denoising level of 100% for this study, which may lead to an artificial image impression for the readers. The impact of different denoising levels on reader acceptance and confidence could be subject to further research in the future.

In conclusion, our findings demonstrate that DLRecon enhances bone depiction in ZTE MRI of the shoulder, enabling increased diagnostic confidence in the assessment of osseous pathologies. These results suggest that DLRecon could add further clinical value to ZTE sequences in shoulder MRI.

## Data Availability

Data are available upon reasonable request.

## Abbreviations

AI: Artificial intelligence
CNN: Convolutional neural network
CNR: Contrast-to-noise ratio
DL: Deep learning
MRI: Magnetic resonance imaging
PACS: Picture archiving and communication system
ROI: Region of interest
SD: Standard deviation
SI: Signal intensity
SNR: Signal-to-noise ratio
SOC: Standard-of-care
ZTE: Zero echo time
2D: Two-dimensional
3D: Three-dimensional
